# A Visual Resolution of Cardiotoxicity: A Case Report of Digoxin-Induced Bidirectional Ventricular Tachycardia

**DOI:** 10.7759/cureus.15134

**Published:** 2021-05-20

**Authors:** Thin Phyu Phyu Aung, Sivacharan Buddhavarapu, Won Jun Park, Cesar Ayala-Rodriguez, Zin Thawdar Oo, Htoo Kyaw

**Affiliations:** 1 Internal Medicine, University of Mandalay, Mandalay, MMR; 2 Cardiology, Brooklyn Hospital Center - Mount Sinai Heart, Brooklyn, USA; 3 Cardiology, Icahn School of Medicine at Mount Sinai, Mount Sinai Hospital, New York, USA; 4 Internal Medicine, Jacobi Medical Center/North Central Bronx Hospital, Bronx, USA; 5 Cardiology, Mount Sinai Hospital, New York, USA

**Keywords:** digoxin, digoxin toxicity, bidirectional ventricular tachycardia, electrocardiogram

## Abstract

Digoxin is rarely used in modern cardiovascular disease management. Therefore, digoxin toxicity has been infrequently encountered and it is paramount to diagnose in a timely fashion. Bidirectional ventricular tachycardia is an unusual arrhythmia wherein every other beat has a different QRS axis as it travels alternately down different conduction pathways. The arrhythmia can be a manifestation of myocarditis, myocardial infarct, Andersen-Tawil syndrome, arrhythmogenic right ventricular cardiomyopathy, catecholaminergic polymorphic ventricular tachycardia, herbal aconite poisoning, and digoxin toxicity. This case illustrates the importance of clinician awareness of rare electrocardiogram (EKG) patterns of digoxin toxicity and visual resolution of fatal arrhythmia with timely treatment.

## Introduction

Digitalis has been used to treat chronic heart failure, arrhythmia, and edematous conditions since ancient times. In contemporary practice, digoxin is used in the treatment of heart failure with reduced ejection fraction and atrial fibrillation despite the lack of mortality benefit [1,2]. Furthermore, clinicians discovered the increased risk of digoxin toxicity due to its narrow therapeutic index [3]. Digoxin toxicity can manifest as a broad spectrum of symptoms such as nausea, vomiting, visual problems, altered mental status, and cardiac arrhythmias, especially in patients with electrolyte imbalance, renal disease, and elderly populations [4,5]. With the occasional use of digoxin, the lack of awareness of digoxin toxicity among new trainees and junior physicians has become epidemic. The following case serves as a reminder to all clinicians regarding the presentation, diagnosis, and treatment of digoxin toxicity.

## Case presentation

A 78-year-old African American female came to the emergency department with epigastric and substernal chest discomfort, nausea, and vomiting, which started about two weeks ago. Now she couldn’t tolerate any food intake with persistent nausea and vomiting for the last few days. Her abdominal discomfort was worse at night and with meals but not relieved by positional change or exertion. She reported a background history of hypertension, coronary artery disease, non-valvular permanent atrial fibrillation (AF), chronic systolic heart failure, biventricular implantable cardioverter-defibrillator (BiV-ICD), and gastro-oesophageal reflux disease. She was taking aspirin 81 mg daily, carvedilol 12.5 mg twice daily, atorvastatin 40 mg daily, apixaban 5 mg twice daily, furosemide 40 mg twice daily, and digoxin 0.25 mg daily.

**Figure 1:**
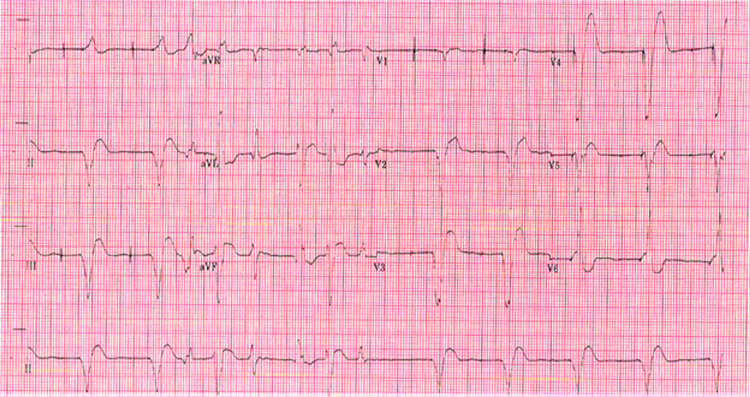
EKG showing a rhythm changes from BDVT to a paced rhythm (noted in the right half of the EKG). BDVT: Bidirectional ventricular tachycardia; EKG: Electrocardiogram.

Upon arrival, the patient’s heart rate was 128/min. Her other vital signs were within normal limits. Physical examination was unremarkable except for tachycardia. The initial laboratory studies showed normal complete blood count, sodium of 133 mmol/L, potassium of 3.4 mmol/L, creatinine of 1.2 mg/dL, and magnesium of 2.5 mmol/L. Initial troponin was noted to be 0.21 mg/dL, and the liver function test was unremarkable. An electrocardiogram (EKG) revealed a wide complex tachycardia with alternating QRS axis, suggestive of bidirectional ventricular tachycardia (BDVT) (Figure 1).

**Figure 2:**
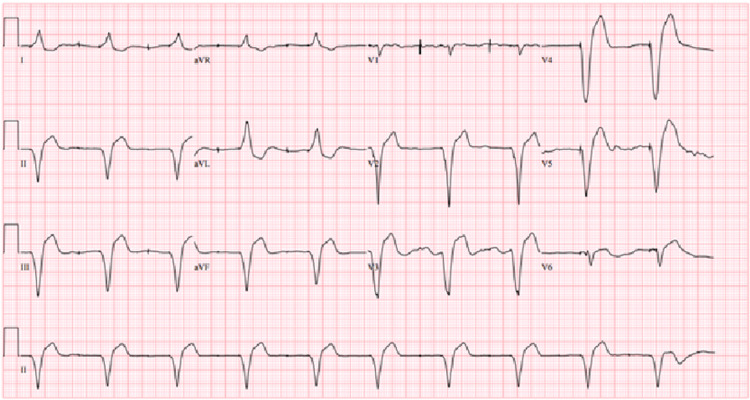
Maintenance of persistently paced rhythm, 24 hours after treatment with DIF. DIF: Digoxin Immune Fab

She was initially treated with IV normal saline 100 ml/hr, IV pantoprazole, IV ondansetron, and potassium supplementation for possible gastritis with dehydration and electrolyte imbalance. However, given significant EKG abnormalities, nausea and vomiting, and regular digoxin intake, digoxin toxicity was entertained as a probable diagnosis. Serum digoxin concentration (SDC) came back 3.6 ng/ml (Normal: 0.5-0.9 ng/ml). The patient was promptly started on Digoxin Immune Fab (DIF) while monitoring the arrhythmia closely. Three hours later, we recorded an event of rhythm conversion from BDVT to a ventricular-paced rhythm. Her symptoms were significantly improved after DIF treatment, and 24-hour telemetry monitoring showed a paced rhythm without any sign of arrhythmia (Figure 2). Upon further inquiry, the causality of digoxin toxicity could be probably due to a combination of electrolyte imbalance, dehydration, and drug interaction between atorvastatin and digoxin, as she was taking medications as prescribed. We stopped digoxin completely, given the higher risk of drug toxicity and continued guideline-directed medical therapy for systolic heart failure. The patient recovered well and was discharged home with follow-up in the cardiology clinic.

## Discussion

Digitalis is one of the oldest medications which continues to be used in modern cardiovascular medicine. Its usage can be dated back to 1785 when Sir William Withering published a textbook about digoxin in treating edematous conditions, irregular heartbeats, and chronic heart failure [1]. After the publication of the Digitalis Investigation Group (DIG) trial in 1997 which failed to show the mortality benefit, there was a significant reduction in digoxin use from 31.4% in late 2001 to 23.5% in late 2004 [1,2]. However, the number of cases with digoxin toxicity for which digoxin antibody was prescribed had significantly increased from 16.2% in 1996 to 30.7% in 2003 [2]. In fact, there is some evidence of increased mortality with SDC >2 ng/ml, and 2010 practice guidelines of the Heart Failure Society of America (HFSA) suggested to maintain SDC <1 ng/ml, preferably 0.7-0.9 ng/ml [6]. Multiple studies have reported conflicting results regarding the mortality impact of digoxin use. The ENGAGE AF TIMI-48 Trial illustrated a statistically significant association between digoxin use and sudden cardiac death in patients with atrial fibrillation with or without heart failure [7]. However, elegant analysis of the post DIG trial done by Davila et al. showed that the inherent weakness of observation on registry data wouldn't be able to provide reliable estimates of digoxin effects [8].

Digoxin's primary mechanism of action is to inhibit the sodium-potassium ATPase pump in the heart's myocardium. Inhibition of this pump causes increased intracellular calcium, resulting in increased myocardial contractility and cardiac output while augmenting cardiac vagal tone [3]. The etiology of digoxin toxicity is multifactorial. The risk of toxicity is increased dramatically in the presence of renal dysfunction, electrolyte imbalance, and elderly population with the presentation of clinical symptoms including anorexia, nausea, vomiting, fatigue, and blurry vision as well as cardiac arrhythmias in the form of paroxysmal atrial tachycardia with block, junctional tachycardia, ventricular tachycardia (VT), premature ventricular contraction, and various degree of heart block [3-5]. In one study conducted by Pita-Fernandez et al., patients presented with nausea (54.8%), asthenia (42.9%), vomiting (33.3%), and anorexia (28.6%) [4].

BDVT is a rare electrocardiographic finding and can be a manifestation of herbal aconite poisoning, myocarditis, myocardial infarct, familial hypokalemia periodic paralysis, catecholaminergic polymorphic VT, and digoxin toxicity [9-12]. The exact mechanism of this arrhythmia has not yet fully understood. According to Baher et al., the probable underlying hypothesis was the presence of two distinct foci with different rate thresholds for ventricular bigeminy cause reciprocal activation, resulting in alternating axis on electrocardiographic measurement [13]. Thus, the importance of early recognition of BDVT couldn't be overemphasized, as it can be life-threatening if not treated on time. Clinician awareness of this unusual EKG presentation is essential as our case's symptoms would have been misinterpreted.

Treatments of digoxin toxicity include adequate fluid resuscitation, gastrointestinal decontamination (within one to two hours of ingestion), digoxin-specific antibody fragments, and electrolyte supplementation [14]. Digoxin-specific antibody fragments consist of a Fab portion of IgG antidigoxin antibodies derived from immunized sheep and bind-free digoxin, thereby forming digoxin-immune fragment complexes, which are renally excreted. DIF is highly effective and safe in digoxin toxicity with life-threatening or hemodynamically unstable arrhythmia, hyperkalemia, and evidence of end-organ dysfunction from hypoperfusion such as renal failure [15, 16]. Continuous monitoring is highly recommended even after treatment with DIF due to the risk of rebound toxicity, which can occur 12-24 hours after treatment [14].

## Conclusions

The use of digoxin in contemporary practice has declined significantly, partly due to advancements in heart failure therapies with mortality benefits, such as angiotensin converting enzyme inhibitor (ACEI)/angiotensin receptor blocker (ARB), angiotensin receptor neprilysin inhibitor (ARNI), beta-blockers, aldosterone blockers, and cardiac resynchronization therapy. However, in a certain subset of heart failure patients with uncontrolled AF and borderline blood pressure, digoxin can be useful due to its little effect on blood pressure and good therapeutic response in AF control. Given the rarity of BDVT occurrence, clinicians need to know the potential impact, presentations, and digoxin toxicity treatment.
